# Potential mechanisms underlying the association between single nucleotide polymorphism (BRAP and ALDH2) and hypertension among elderly Japanese population

**DOI:** 10.1038/s41598-020-71031-9

**Published:** 2020-08-25

**Authors:** Yuji Shimizu, Kazuhiko Arima, Yuko Noguchi, Shin-Ya Kawashiri, Hirotomo Yamanashi, Mami Tamai, Yasuhiro Nagata, Takahiro Maeda

**Affiliations:** 1 Department of Community Medicine, Nagasaki University Graduate School of Biomedical Sciences, Nagasaki-shi, Sakamoto 1-12-4, Nagasaki, 852-8523 Japan; 2 Department of Cardiovascular Disease Prevention, Osaka Center for Cancer and Cardiovascular Disease Prevention, Osaka, Japan; 3 Department of Public Health, Nagasaki University Graduate School of Biomedical Sciences, Nagasaki, Japan; 4 Department of General Medicine, Nagasaki University Graduate School of Biomedical Sciences, Nagasaki, Japan; 5 Department of Island and Community Medicine, Nagasaki University Graduate School of Biomedical Sciences, Nagasaki, Japan; 6 Center for Comprehensive Community Care Education, Nagasaki University Graduate School of Biomedical Sciences, Nagasaki, Japan

**Keywords:** Biomarkers, Cardiology, Health care, Risk factors

## Abstract

Minor allele frequency (MAF) of rs3782886 (BRAP) and rs671 (ALDH2) are reported to be inversely associated with blood pressure. Another study revealed that hematopoietic activity which is evaluated by reticulocytes could influenced on hypertension status partly by indicating activity of endothelial maintenance. Therefore, to evaluate the association between genetic factor and hypertension, influence of hematopoietic activity should be considered. A multi-faced analysis was performed in a simple general elderly population model (1,313 older Japanese aged 60–98 years). Participants were stratified by median values of reticulocytes (5.21 × 10^4^ cells/μL for men and 4.65 × 10^4^ cells/μL for women). Independent of known cardiovascular risk factors, MAF of rs3782886 and rs671 are significantly inversely associated with hypertension for participants with high hematopoietic activity (high reticulocytes level) (fully adjusted odds ratio (ORs) were 0.72 (0.55, 0.96) for rs3782886 and 0.72 (0.54, 0.96) for rs671) but not for low reticulocytes count (the corresponding values were 1.05 (0.79, 1.39) and 1.08 (0.81, 1.45), respectively). Hematopoietic activity evaluated by reticulocytes levels could influence on the association between single nucleotide polymorphism (rs3782886 and rs671) and hypertension. Those results were efficient tool to clarify the part of the mechanism that underlying the association between genetic factor and hypertension.

## Introduction

Atherosclerosis is generally considered to be positively associated with hypertension^1^. A single nucleotide polymorphism (SNP) (rs3782886) in breast cancer suppressor BRCA1**-**related associated protein (BRAP) has been associated with the risk of developing myocardial infarction^2^. Another study reported that BRAP activates inflammatory cascades, increasing the risk of carotid atherosclerosis^3^. Even though the minor allele frequency (MAF) of rs3782886, and aldehyde dehydrogenase 2 (ALH2) polymorphism rs671 are significantly inversely associated with both systolic and diastolic blood pressure^4^.

Since strong linkage disequilibrium (LD) values between rs3782886 and rs671 have been reported^5^, rs671 could have same the characteristics of rs3782886. However, the mechanism underlying the association between hypertension and these genetic factors (rs3782886 and rs671) have not yet been elucidated.

Atherosclerosis is involved in aggressive endothelial repair. Therefore, the favorable association of genetics factor (rs3782886 and rs671) with hypertension may be caused by stimulating endothelial repair.

Additionally, several studies have demonstrated a close association between bone marrow activity (hematopoietic activity) and endothelial maintenance^6, 7^. We have showed in a previous study that active endothelial repair, represented by high levels of hematopoietic (CD34-positive) stem cells, is positively associated with atherosclerosis but could have beneficial effect on prevention of hypertension among elderly participants^8^.

Hematopoietic bone marrow activity declines with age^9^ and aging is also a well-known cause of endothelial injury^10, 11^. Since hematopoietic activity also influences reticulocyte levels, high levels of reticulocytes among older participants may indicate a high capacity of endothelial maintenance. Therefore, the levels of reticulocytes could play a role in the association between genetic factors (rs3782886 and rs671) and hypertension.

Furthermore, platelet activation is involved in the initial mechanism of an endothelial repair^12, 13^ and a platelet count indicates the activity of an endothelial repair^1, 14^. Therefore, evaluating the number of platelets may be an efficient tool to determine the mechanism underlying the association between genetic factors (rs3782886 and rs671) and hypertension.

An extensive prevalence of SNPs should have the same beneficial effect on the participants’ daily activities, rather than impose a disadvantage. Therefore, determining the mechanism by which genetic characteristics prevent hypertension inducing progression of atherosclerosis could provide novel knowledge to elaborate strategies for risk estimation and prevention of hypertension.

To determine the potential mechanism underlying the association between the genetic factors (rs3782886 and rs671) and hypertension, we conducted a cross-sectional study comprised of 1,313 elderly Japanese individuals (aged 60–98) who had previously participated in a general health check-up, in 2017.

## Methods

### Study population

The methods that relates to present risk survey including genetic factor also have been described elsewhere^1, 14–16^.

The study population was comprised of 1,401 Japanese residents (504 men and 897 women) aged between 60 and 99 years old from Goto City (western Japan) who had undergone an annual medical check-up in 2017.

Participants without platelet data (n = 2) or SNP data (n = 1) were excluded from the study population. Additionally, to control for the influence of chronic disease, participants with hypo-nutrition (BMI ≤ 18.0 kg/m^2^) (n = 85) were also excluded. The remaining participants, comprising 1,313 (494 men and 819 women) with a mean age of 72.9 (standard deviation (SD): 7.3; range: 60–98), were enrolled in the study.

All of the procedures involving human participants in this study were performed in accordance with the ethical standards of the institution research committee and the 1964 Helsinki Declaration, and its later amendments for comparable ethical standards. Written consent forms in Japanese were made available to ensure the comprehensive understanding of the study objectives, and informed consent was provided by the all participants. Ethics Committee of Nagasaki University Graduate School of Biomedical Sciences (project registration number: 14051404) approved this study.

### Data collection and laboratory measurements

Specially trained interviewers were tasked with obtaining the medical histories and habitual statuses of the participants. The body weight and height were measured using an automatic body composition analyzer (BF-220; Tanita, Tokyo, Japan), from which the body mass index (BMI; kg/m^2^) was calculated. Systolic (SBP) and diastolic blood pressure (DBP) were recorded at rest. Hypertension was defined as systolic blood pressure ≥ 140 mmHg and/or diastolic blood pressure ≥ 90 mmHg and/or taking anti-hypertensive medication.

Fasting blood samples were collected in a heparin sodium tube, an EDTA-2K tube, and a siliconized tube.

The quantities of platelets, red blood cells (RBC), and reticulocytes in the samples from the EDTA-2K tube were measured using an automated procedure at SRL, Inc. The levels of reticulocytes were determined using the following formula: reticulocytes (×10^4^ cells/μL) = (reticulocytes, ‰) × RBC (×10^4^ cells/μL)/1,000 (for men: 5.37 ± 2.01 [×10^4^ cells/μL]; for women: 4.78 ± 1.56 [×10^4^ cells/μL]). The concentrations of triglyceride (TG), HDL-cholesterol (HDL-C), hemoglobin A1c (HbA1c), and creatinine were measured using the standard laboratory procedures. All measurements were performed at SRL, Inc. (Tokyo, Japan). The glomerular filtration rate (GFR) was estimated using a recently adapted established method introduced by a working group of the Japanese Chronic Kidney Disease Initiative^17^, which yielded an estimate of GFR (mL/min/1.73 m^2^) = 194 × (serum creatinine (enzyme method))^−1.094^ × (age)^−0.287^ × (0.739 for women).

The genomic DNA was extracted from 2 mL of whole peripheral blood using the Gene Prep Star NA-480 (Kurabo Industries Ltd., Osaka, Japan), which was genotyped for SNP rs3782886 (BRAP on chromosome 12q24.12) and rs671 (ALDH2 on chromosome 12q24.2) using the TaqMan method with a LightCycler 480 (Roche Diagnostics, Basel, Switzerland). Expected numbers and percentages were calculated. Deviations from the Hardy–Weinberg equilibrium were evaluated via χ^2^ test for each allele (rs3782886 and rs671).

### Statistical analysis

The study population was classified by reticulocyte levels (high and low) according to the median values (5.21 × 10^4^ cells/μL for men and 4.65 × 10^4^ cells/μL for women).

The characteristics of the study population in relation to the reticulocyte levels were expressed as the mean ± standard deviation (SD) for the contentious variables except for TG, and as prevalence for the medication status and habitual status. Since TG showed a skewed distribution, the characteristics of the study population were expressed as median [first quartile and third quartile], followed by a logarithmic transformation. Differences between mean values or proportional values of monitored characteristics were analyzed in relation to reticulocyte levels. Significant differences were evaluated using t-test for continuous variables and χ^2^ test for proportional data.

Logistic regression models were used to calculate the odds ratios (OR) and 95% confidence intervals (CI) were used to determine associations between hypertension and platelets, as well as between hypertension and reticulocyte levels.

The numbers of platelets and reticulocytes in relation to the genotype (rs3782886 and rs671) by reticulocyte levels were also calculated.

In addition, OR and 95% CI were also calculated using a logistic regression model to determine the influence of SNP (rs3782886 and rs671) on hypertension by the levels of reticulocytes.

Two different approaches were used to make adjustments for confounding factors. First, one model was adjusted only for sex and age (Model 1). For the second model (Model 2), we included several other potential confounding factors, namely BMI (kg/m^2^), alcohol consumption (never drinker, former drinker, current drinker [23–45 g/week, 46–68 g/week, ≥ 69 g/week]), smoking status (never, former, current), TG (mg/dL), HDL-C (mg/dL), HbA1c (%), and GFR (mL/min/1.73 m^2^).

As Japanese men are known to have high rates of drinking, while Japanese women have low rates of drinking^18^, to evaluate the influence of SNP (rs3782886 and rs671) on the status of individual who had never had a drink (never drinker), sex-specific analyses were performed.

Furthermore, to evaluate the influence of drinking status (never drinker) on platelet and reticulocyte count, sex-adjusted value of platelet and reticulocyte count in relation to drinking status stratified by reticulocyte levels were calculated by using covariance analysis.

All statistical analyses were performed using the SAS system for Windows (version 9.4; SAS Inc., Cary, NC). P-values < 0.05 were regarded as statistically significant.

## Results

### Characteristics of study population by reticulocyte levels

Characteristics of study population by reticulocyte levels are shown in Table 1.

**Table 1:** Characteristics of study population by reticulocyte levels.

	Reticulocyte levels		*p*
	Low	High	
No. of participants	656	657	
Men, %	37.7	37.6	0.983
Age, years	73.7 ± 7.7	72.2 ± 6.7	< 0.001
SBP, mmHg	134 ± 17	135 ± 17	0.075
DBP, mmHg	73 ± 10	75 ± 11	< 0.001
Anti-hypertensive medication, %	39.6	47.5	0.004
BMI, kg/m^2^	22.2 ± 2.6	23.8 ± 3.1	< 0.001
Current drinker, %	31.9	39.6	0.004
Current smoker, %	7.0	8.1	0.470
TG, mg/dL	85 [65, 113]^a^	102 [74, 139]^a^	< 0.001^b^
HDL-C, mg/dL	62 ± 15	61 ± 15	0.070
HbA1c, %	5.7 ± 0.5	5.8 ± 0.6	0.035
GFR, mL/min/1.73m^2^	68.5 ± 14.3	68.7 ± 14.0	0.831
Plt, × 10^4^ /μL	22.5 ± 5.1	22.7 ± 4.9	0.453

Values: mean ± standard deviation.

SBP: Systolic blood pressure. DBP: Diastolic blood pressure. BMI: Body mass index. TG: Triglycerides. HDL-C: HDL-cholesterol. GFR: Glomerular filtration rate. Plt: Platelets.

^a^Values are median [first quartile, third quartile].

^b^Logarithmic transformation was used for evaluating p.

Compared to participants with low reticulocyte levels, the participants with high reticulocyte levels showed significantly higher values of DBP, anti-hypertensive medication use, BMI, current drinker, TG, and HbA1C, but a significantly lower age.

### Association between hypertension and platelet among total participants

Platelets play an important role in endothelial repair^13^. Moreover, platelet count indicates endothelial repair activity^14^. As such, we evaluated the association between hypertension and platelet count for the total of the participants (Table 2). Independent from known cardiovascular risk factors, platelets were significantly positively associated with hypertension.

**Table 2:** Odds ratios (ORs) and 95% confidence intervals (CIs) for hypertension in relation to platelets and reticulocytes among total participants.

	Platelet tertiles			*p* for trend	1 SD increment of platelets
	T1 (low)	T2	T3 (high)		
No. of participants	441	432	440		
No. of cases (%)	244 (55.3)	270 (62.5)	279 (63.4)		
Model 1	1.00	1.47 (1.11, 1.94)	1.61 (1.22, 2.14)	< 0.001	1.23 (1.10, 1.39)
Model 2	1.00	1.38 (1.02, 1.85)	1.56 (1.16, 2.09)	0.004	1.22 (1.08, 1.38)

	Reticulocyte tertiles			*P* for trend	1 SD increment of reticulocytes
	T1 (low)	T2	T3 (high)		
No. of participants	437	438	438		
No. of cases (%)	239 (54.7)	264 (60.3)	290 (66.2)		
Model 1	1.00	1.31 (0.99, 1.73)	1.88 (1.42, 2.50)	< 0.001	1.44 (1.26, 1.64)
Model 2	1.00	1.09 (0.81, 1.45)	1.21 (0.89, 1.65)	0.222	1.19 (1.03, 1.36)

Model 1: Adjusted only for sex and age. Model 2: Adjusted further for sex and age, alcohol consumption (never drinker, former drinker, current drinker [23–45 g/week, 46–68 g/week, ≥ 69 g/week]) , smoking status (never smoker, former smoker, current smoker), body mass index, HDL-cholesterol, triglycerides, HbA1c, and GFR. Platelet tertiles: For men < 19.6 × 10^4^/μL for T1, 19.6–23.5 × 10^4^/μL for T2, > 23.5 × 10^4/^μL for T3. For women < 20.9 × 10^4^/μL for T1, 20.9–24.6 × 10^4^/μL for T2, > 24.6 × 10^4^/μL for T3. The 1 standard deviation (SD) increment of platelets was 5.2 × 10^4^/μL for men and 4.8 × 10^4^/μL for women. Reticulocyte tertiles: For men, < 4.464 × 10^4^ cells/μL for T1, 4.464–5.796 × 10^4^/cells/μL for T2, > 5.796 × 10^4^/μL for T3. For women, < 4.0 × 10^4^ cells/μL for T1, 4.0–5.29 × 10^4^ cells/μL for T2, > 5.29 × 10^4^ cells/μL for T3. The increments of 1 standard deviation (SD) in reticulocytes are 2.0 × 10^4^ cells/μL for men and 1.6 × 10^4^ cells/μL for women.

### Association between hypertension and reticulocyte levels among total participants

Table 2 shows the association between hypertension and reticulocyte levels for the total participants. Independent from known cardiovascular risk factors, the reticulocyte levels were significantly positively associated with hypertension.

### Association between hypertension and genotype of rs3782886 by reticulocyte levels

The OR and 95% CI of hypertension for the rs3782886 genotype based on the levels of reticulocytes are shown in Table 3. Despite no significant associations being found between hypertension and MAF of rs3782886 with low levels of reticulocytes, a significantly inverse association was observed in participants with high levels of reticulocytes. We also found that the interactions between the reticulocyte levels had a significant effect on the association between rs3782886 and hypertension, with a fully adjusted p-value (Model 2) of 0.0499. Despite no significant associations being found between platelet count and MAF of rs3782886 with low levels of reticulocytes, a significantly positive association was observed with high levels of reticulocytes. In addition, for both low and high reticulocytes, no significant association between reticulocyte levels and the MAF of the rs3782886 genotype was observed.

**Table 3:** Odds ratios (OR) and 95% confidence intervals (CI) for hypertension in relation to rs3782886 genotype by reticulocyte levels.

	Major homo (A/A)	Hetero (A/G)	Minor homo (G/G)	*p* for trend	MAF
**Low reticulocyte**					
No. of participants (%)	380 (57.9)	232 (35.4)	44 (6.7)	≥ 0.05*	
No. of expectations (%)	375.02 (57.2)	241.95 (36.9)	39.02 (5.9)		
No. of cases (%)	213 (56.1)	128 (55.2)	24 (54.5)		
Platelets, ×10^4^ /μL	22.3 ± 5.2	22.8 ± 4.7	23.0 ± 5.6	0.480	–
Reticulocyte, ×10^4^ cells/μL	3.7 ± 0.7	3.8 ± 0.7	3.7 ± 0.8	0.790	–
Model 1	1.00	0.98 (0.70, 1.39)	0.95 (0.50, 1.81)	0.872	0.98 (0.76, 1.27)
Model 2	1.00	1.13 (0.78, 1.65)	0.96 (0.48, 1.92)	0.753	1.05 (0.79, 1.39)
**High reticulocyte**					
No. of participants (%)	395 (60.1)	208 (31.7)	54 (8.2)	≥ 0.05*	
No. of expectations (%)	379.00 (57.7)	240.01 (36.5)	38.00 (5.8)		
No. of cases (%)	271 (68.6)	129 (62.0)	28 (51.9)		
Platelets, ×10^4^ /μL	22.2 ± 4.8	23.5 ± 4.9	23.7 ± 5.0	0.005	–
Reticulocyte, ×10^4^ cells/μL	6.3 ± 1.8	6.2 ± 1.1	6.1 ± 1.0	0.407	–
Model 1	1.00	0.72 (0.50, 1.03)	0.49 (0.27, 0.88)	0.007	0.71 (0.55, 0.91)
Model 2	1.00	0.71 (0.48, 1.06)	0.53 (0.28, 1.02)	0.025	0.72 (0.55, 0.96)

Model 1: adjusted only for sex and age. Model 2: Adjusted further for sex, age, alcohol consumption (never drinker, former drinker, current drinker [23–45 g/week, 46–68 g/week, ≥ 69 g/week]) , smoking status (never smoker, former smoker, current smoker), body mass index, HDL-cholesterol, triglycerides, HbA1c, and GFR. The hypertension is defined as systolic blood pressure ≥ 140 mmHg and/or diastolic blood pressure ≥ 90 mmHg and/or taking anti-hypertensive medication. MAF: minor allele frequency.

*Hardy–Weinberg equilibrium p-values.

### Association between hypertension and genotype of rs671 by reticulocyte levels

Those associations for rs3782886 were essentially the same as those for rs671, as shown in Table 4. Despite no significant association being found between hypertension and the MAF of rs671 with low levels of reticulocytes, a significantly inverse association was observed with high levels of reticulocytes. We also found that the interactions between the reticulocyte levels had a significant effect on rs671 and hypertension, with a fully adjusted p-value (Model 2) of 0.035.

**Table 4:** Odds ratios (OR) and 95% confidence intervals (CI) for hypertension in relation to rs671 genotype by reticulocyte levels.

	Major homo (G/G)	Hetero (G/A)	Minor homo (A/A)	*p* for trend	MAF
**Low reticulocyte**					
No. of participants (%)	394 (60.1)	224 (34.1)	38 (5.8)	≥ 0.05*	
No. of expectations (%)	390.30 (59.5)	231.40 (35.3)	34.30 (5.2)		
No. of cases (%)	219 (55.6)	125 (55.8)	21 (55.3)		
Platelets, ×10^4^ /μL	22.3 ± 5.3	22.8 ± 4.5	23.0 ± 5.9	0.515	–
Reticulocyte, ×10^4^ cells/μL	3.7 ± 0.7	3.8 ± 0.7	3.7 ± 0.8	0.876	–
Model 1	1.00	1.02 (0.72, 1.44)	0.98 (0.49, 1.94)	0.983	1.00 (0.77, 1.31)
Model 2	1.00	1.16 (0.80, 1.69)	1.04 (0.50, 2.15)	0.588	1.08 (0.81, 1.45)
**High reticulocyte**					
No. of participants (%)	413 (62.9)	195 (29.7)	49 (7.5)	≥ 0.05*	
No. of expectations (%)	396.67 (60.4)	227.67 (34.7)	32.67 (5.0)		
No. of cases (%)	281 (68.0)	123 (63.1)	24 (49.0)		
Platelets, ×10^4^ /μL	22.2 ± 4.8	23.5 ± 4.9	24.0 ± 5.1	0.002	–
Reticulocyte, ×10^4^ cells/μL	6.3 ± 1.8	6.1 ± 1.1	6.1 ± 1.0	0.249	–
Model 1	1.00	0.79 (0.55, 1.14)	0.46 (0.25, 0.85)	0.012	0.72 (0.56, 0.93)
Model 2	1.00	0.75 (0.50, 1.12)	0.50 (0.25, 0.98)	0.027	0.72 (0.54, 0.96)

Model 1: adjusted only for sex and age. Model 2: Adjusted further for sex, age, alcohol consumption (never drinker, former drinker, current drinker [23–45 g/week, 46-68 g/week, ≥ 69 g/week]) , smoking status (never smoker, former smoker, current smoker), body mass index, HDL-cholesterol, triglycerides, HbA1c, and GFR. The hypertension is defined as systolic blood pressure ≥ 140 mmHg and/or diastolic blood pressure ≥ 90 mmHg and/or taking anti-hypertensive medication.

MAF: minor allele frequency.

*Hardy–Weinberg equilibrium p-values.

Despite no significant association being found between the platelet count and the MAF of rs671 with low levels of reticulocytes, a significantly positive association was observed for high levels of reticulocytes. In addition, for both low and high levels of reticulocytes, no significant association between reticulocytes and MAF of rs671 was observed.

### Sex-specific association between never drinker and rs3782886 and rs671 genotypes

The sex-specific associations between MAF of SNP (rs3782886 and rs671) and never drinker are shown in Tables 5, 6. Both the MAF of rs3782886 and rs671 showed a significantly positive association in participants classified as never drinkers.

**Table 5:** Odds ratios (OR) and 95% confidence intervals (CI) for never drinker in relation to rs3782886 genotype.

	rs3782886				
	Major homo (A/A)	Hetero (A/G)	Minor homo (G/G)	*p* for trend	MAF
**Men**					
No. of participants (%)	282 (57.1)	172 (34.8)	40 (8.1)	< 0.05*	
No. of expectations (%)	274.14 (55.5)	187.70 (38.0)	32.14 (6.5)		
No. of cases (%)	34 (12.1)	72 (41.9)	31 (77.5)		
Age-adjusted	1.00	5.28 (3.29, 8.46)	26.45 (11.52, 60.71)	< 0.001	5.20 (3.63, 7.45)
**Women**					
No. of participants (%)	493 (60.2)	268 (32.7)	58 (7.1)	≥ 0.05*	
No. of expectations (%)	480.01 (58.6)	293.98 (35.9)	45.01 (5.5)		
No. of cases (%)	363 (73.6)	238 (88.8)	56 (96.6)		
Age-adjusted	1.00	2.93 (1.90, 4.52)	10.48 (2.51, 43.70)	< 0.001	3.01 (2.06, 4.39)

MAF: minor allele frequency.

*Hardy–Weinberg equilibrium p-values.

**Table 6:** Odds ratios (OR) and 95% confidence intervals (CI) for never drinker in relation to rs671 genotype.

	rs671				
	Major homo (G/G)	Hetero (G/A)	Minor homo (A/A)	*p* for trend	MAF
**Men**					
No. of participants (%)	296 (59.9)	164 (33.2)	34 (6.9)	< 0.05*	
No. of expectations (%)	289.24 (58.6)	177.52 (35.9)	27.24 (5.5)		
No. of cases (%)	38 (12.8)	71 (43.3)	28 (82.4)		
Age-adjusted	1.00	5.21 (3.28, 8.27)	33.09 (12.79, 85.61)	< 0.001	5.47 (3.78, 7.92)
**Women**					
No. of participants (%)	511 (62.4)	255 (31.1)	53 (6.5)	≥ 0.05*	
No. of expectations (%)	497.78 (60.8)	281.44 (34.4)	39.78 (4.9)		
No. of cases (%)	376 (73.6)	229 (89.8)	52 (98.1)		
Age-adjusted	1.00	3.27 (2.07, 5.16)	20.05 (2.74, 146.91)	< 0.001	3.49 (2.32, 5.25)

MAF: minor allele frequency.

*Hardy–Weinberg equilibrium p-values.

### Sex-adjusted reticulocyte count in relation to never-drinker status

Since gender determined a statistically significant difference (p < 0.001) in reticulocyte levels between (mean ± SD) were for men (5.37 ± 2.01 × 10^4^ cells/μL, n = 494) and women (4.78 ± 1.56 × 10^4^ cells/μL, n = 819), adjustment of data by sex was performed to evaluate the relationship between reticulocyte levels and never-drinker status. Never drinkers displayed significantly lower reticulocyte levels than non-never drinkers (*p* = 0.006), with a sex-adjusted reticulocyte count (mean ± standard error (SE)) of 4.88 ± 0.07 × 10^4^ cells/μL for never drinkers (n = 794) and 5.19 ± 0.08 × 10^4^ cells/μL for non-never drinkers (n = 519).

### Sex-adjusted platelet count in relation to never-drinker status

Since gender determined a statistically significant difference (p < 0.001) in platelet levels (mean ± SD) between men (23.1 ± 4.8 × 10^4^ /μL) and women (21.9 ± 5.2 × 10^4^ /μL), adjustment of data by sex was performed to evaluate the relationship between platelet levels and never-drinker status. We did not find any significant difference in platelet levels between never and non-never drinkers (*p* = 0.059); the sex-adjusted platelet count (mean ± SE) was 22.9 ± 0.2 × 10^4^ /μL for never drinkers and 22.3 ± 0.2 × 10^4^ /μL for non-never drinkers.

### Sex-adjusted platelet counts in relation to never-drinker status by reticulocyte levels

Among the participants with high levels of reticulocytes, never-drinkers showed significantly higher platelet counts than those in the non-never drinkers (*p* = 0.046); the sex-adjusted platelet counts (mean ± SE) were 23.1 ± 0.3 for never drinkers (n = 369) and 22.2 ± 0.3 for non-never drinkers (n = 288). Among the participants with low reticulocyte levels, no significant association was observed (*p* = 0.444); the corresponding values were 22.7 ± 0.3 for never-drinkers (n = 425) and 22.3 ± 0.4 for non-never drinkers (n = 231).

### Present study population in relation to the Hardy–Weinberg equilibrium

An established Hardy–Weinberg equilibrium for both genetic factors (rs3782886 and rs671) was observed only in men (Tables 5, 6).

## Discussion

In terms of the major findings presented in this study, the MAF of rs3782886 and rs671 were found to be significantly inversely associated with hypertension only in individuals with high levels of hematopoietic activity (high reticulocyte levels).

In addition to that the MAF of rs3782886 and rs671 were found to be positively associated with the platelet count only in individuals with high reticulocyte levels. Furthermore, the status of never-drinkers showed significantly higher platelet counts than those in non-never drinkers, only in individuals with high reticulocyte levels, whereas the MAF of rs3782886 and rs671 was significantly and positively associated with the status of a never-drinker. Since ethanol directly attenuates the activation of platelets^19^, which plays an important role in endothelial repair^13, 14^, these associations provide an efficient tool to clarify the influence of genetic factors on hypertension in the Japanese population.

**Figure 1:**
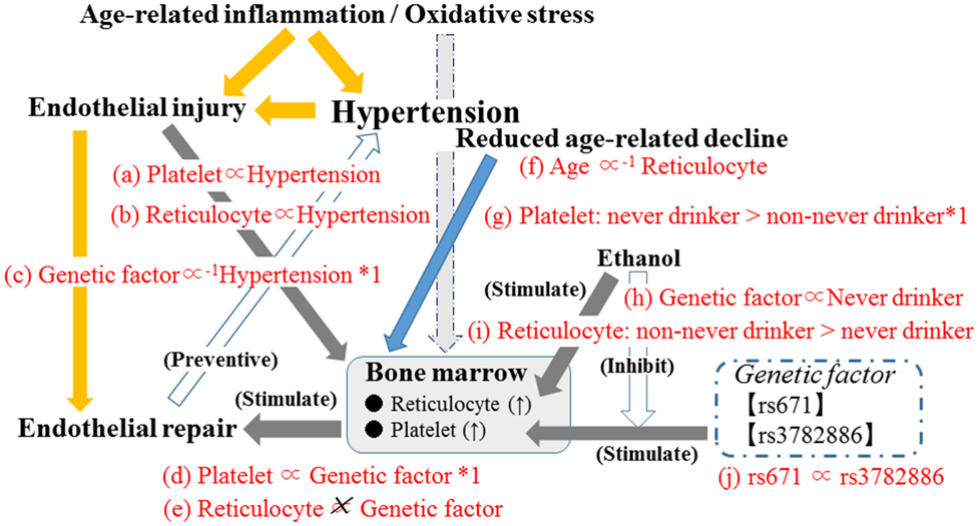
Potential mechanism underlying present results. Associations shown in red (**a**–**j**) were observed in the present study. Genetic factor: minor allele frequency of rs671 and rs3782886. *1: Observed only among participants with high reticulocyte.

A previous study conducted on a Japanese found that the MAF of rs3782886 and rs671 were significantly inversely associated with both systolic and diastolic blood pressure^4^. In the present study, we found further evidence that the MAF of these genotypes were significantly inversely associated with hypertension only in participants with high levels reticulocytes (Tables 3, 4, Fig. 1c). However, the background mechanism responsible for these associations has yet to be fully elucidated.

A summary of the potential mechanisms underlying our results is provided in Fig. 1. Genetic factors stimulate platelet production, which contributes to endothelial repair. Moreover, an age-related reduction in bone marrow activity, which is associated with low levels of reticulocytes, may reduce the impact of this genetic influence.

Recently, a close connection between the bone metabolism and the activity of vascular maintenance has been found^7^. Furthermore, an age-related reduction in bone marrow activity (hematopoietic activity) is known to cause anemia in the elderly^20^. In present study, individuals with high reticulocyte level shows significantly younger than that of individuals with low reticulocyte level (Table 1, Fig. 1f). And reticulocyte is revealed to be significantly positively associated with hypertension (Table 2, Fig. 1b) as like previous our study^15^. Since hypertension injures endothelium strongly, lower levels of reticulocytes among the elderly indicate a lower capacity for vascular maintenance. Even though genetic factors have a beneficial effect on preventing hypertension by stimulating vascular maintenance activity, in the present study, participants with low levels of hematopoietic activity did not appear to benefit from this (Tables 3, 4, Fig. 1c). In other words, the hematopoietic activity evaluated by the reticulocyte levels may influence the association between genetic factors and hypertension.

The platelet has been reported to contribute to endothelial repair^13^. Our previous study found that platelet levels were positively associated with hypertension^1^ and that the platelet count could serve as an indicator of endothelial repair^14^. In the present study, platelet count was found to be significantly positively associated with hypertension (Table 2, Fig. 1a). Therefore, the platelet count may act as an indicator of endothelial repair activity, since endothelial injury caused by hypertension activates endothelial repair.

On the other hand, the results presented here only showed a significantly positive association between rs3782886 and platelet counts in participants with high levels of reticulocytes (Table 3, Fig. 1d), whereas the MAF of rs3782886 was found to be significantly inversely associated hypertension in these participants (high levels of reticulocytes) (Table 3, Fig. 1c). These results seem ambivalent associations since we also found significant positive associations between platelet count and hypertension in the general study population (Table 2, Fig. 1a). This ambivalent association may be the result of different pathways increasing the platelet levels. Endothelial injury caused by hypertension stimulates platelet production, which results in positive association between these two factors. On the other hand, genetic factors stimulate endothelial repair by inducing the production of platelets, which has a beneficial effect on preventing hypertension, since endothelial dysfunction has a bidirectional relationship with hypertension^1^. In a previous study, were found that the MAF of rs3782886 (BRAP) was significantly positively associated with a high platelet count, which is associated with hypertension^16^, supports this mechanism. In addition, as in our previous study^15^, here, we found a significantly positive association between reticulocyte levels and hypertension (Table 2, Fig. 1b), while no significant association was found between reticulocyte levels and genetic factors (rs3782886 and rs671) (Tables 3, 4, Fig. 1e), also supporting this mechanism; hypertension related endothelial injury caused by process of aging such as low grade inflammation and increased oxidative stress^10, 11^ stimulates the production of reticulocytes, while genetic factors do not stimulate reticulocytes production.

A potential biochemical mechanism that may underlie the association between the rs3782886 genotype and platelet count is the activation of the NF-κB pathway. A higher expression of the BRAP minor allele is associated with an increased risk of atherosclerosis, possibly by heightening the degree of inflammation via the activation of the NF-κB pathway^3, 21^. Since the activation of the NF-κB pathway may also promote platelet activation proteins^22^, the NF-κB pathway may influence the association between rs3782886 and platelet count. An increased platelet count stimulates endothelial repair activity^14^.

Strong linkage disequilibrium (LD) values have been reported between rs3782886 and rs671^5^. In the present study, we found a significantly positive correlation between the MAF of rs671 and rs3782886 (Fig. 1j). The simple correlation coefficient (r) of these genotypes were r = 0.94 (p < 0.001) for men and r = 0.94 (p < 0.001) for women. Therefore, we found essentially the same associations as for the rs671 genotype (Tables 3, 4, 5, 6, Fig. 1c,d,e,h). Similar to a previous study^23^, we found that rs3782886 was strongly associated with the status of the never drinkers (Table 5, Fig. 1h). From an anthropological point of view, an extensive prevalence of SNPs should have the same beneficial effect on the participants’ daily activities, rather than impose a disadvantage. Ethanol induces oxidative stress^24^ that might stimulate reticulocyte production. In the present study, we found that non-never drinkers displayed significantly higher reticulocyte levels than never drinkers (Fig. 1i). In addition, acute ethanol exposure dramatically inhibits NF-κB activation^25^. And rs3782886 exists primarily for the activation of endothelial repair via activating the NF-κB pathway. Furthermore, another study reported that ethanol directly attenuates platelet activation and has a significant endothelial cell-mediated effect on selected markers of atherosclerosis *in vitro*^19^. Therefore, avoiding exposure to ethanol is highly beneficial for individuals with the non-major genotype of rs3782886. In the present study, never-drinkers showed significantly higher platelet counts than those in the non-never drinkers with high reticulocyte levels, but not for those individuals with low reticulocyte levels (Fig. 1g).

Hypertension, which is widely found in Japan, is a known risk factor of stroke^26^. Japanese individuals are characterized by higher rates of stroke than myocardial infarction^27^. Even SNPs in BRAP have been reported to be associated with the risk of myocardial infarction^2^, but not with stroke^28^. As such, genetic factor has the potential to have a strong beneficial influence on preventing hypertension, especially in the Japanese population, which has a higher risk of stroke. In the present study, among participants carrying the minor rs3782886 allele, never drinkers were more represented among women than among men; indeed, 88.8% of heterozygous and 96.6% of homozygous women for rs3782886 were never drinkers, while for men the corresponding values were 41.9% and 77.5%. Therefore, the beneficial influence of the present genetic factor might be stronger for women than men. Since the present study population is composed of elderly Japanese, such beneficial influence on lifespan might result in a disrupted Hardy–Weinberg equilibrium in women but not in men. Since the present genetic factor might exert its beneficial influence by activating endothelial repair, further investigations with a long-term follow-up study of oxidative stress is necessary to verify this hypothesis.

This study contains several strengths. Firstly, this study is the first to demonstrate the influence of hematopoietic activity on the association between genetic factors and hypertension. Unlike previous epidemiological studies, this study used multi-faceted analyses to determine potential mechanisms underlying our main findings. Furthermore, the Japanese population is known to have a high prevalence for the minor allele of rs671, which is associated with a low tolerance to ethanol exposure^29^. This study is also the first to reports the potential mechanism for why the Japanese population expresses genetic factors that are unfavorable to ethanol exposure.

This study has some limitations that warrant consideration. Firstly, the activation of the NF-κB protein may have influenced our present results, as no data concerning NF-κB protein was available. Further studies taking into account data for the activity of NF-κB protein will be necessary. In conjunction with the circulating CD34-posistive cell levels, the platelet count plays an important role in endothelial repair^1, 14^. Therefore, the levels of circulating CD34-positive cell may also act as a determining factor in the present analysis. However, due to the difficulty of measuring CD34-posistive cells in a general health check-up, no data on the levels of circulating CD34-posistive cells were included in this study.

In conclusion, the MAF of rs3782886 and rs671 are only significantly inversely associated with hypertension and significantly and positively associated with the platelet count in individuals with high levels of hematopoietic activity (high reticulocyte levels). Furthermore, the status of never-drinkers showed significantly higher platelet counts than those in non-never drinkers with high reticulocyte levels, whereas the MAF of rs3782886 and rs671 was significantly and positively associated with the status of a never-drinker. Since ethanol directly attenuates platelet activation^19^ these associations represented efficient tool to clarify the genetic factors that influence hypertension in the Japanese population.
